# Role of Artificial Intelligence and Machine Learning in Prediction, Diagnosis, and Prognosis of Cancer

**DOI:** 10.7759/cureus.31008

**Published:** 2022-11-02

**Authors:** Kritika Gaur, Miheer M Jagtap

**Affiliations:** 1 Pathology, Jawaharlal Nehru Medical College, Datta Meghe Institute of Medical Sciences, Wardha, IND

**Keywords:** increase survival rates, cancer prognosis, cancer diagnosis, cancer prediction, deep learning, machine learning, artificial intelligence

## Abstract

Cancer is one of the most devastating, fatal, dangerous, and unpredictable ailments. To reduce the risk of fatality in this disease, we need some ways to predict the disease, diagnose it faster and precisely, and predict the prognosis accurately. The incorporation of artificial intelligence (AI), machine learning (ML), and deep learning (DL) algorithms into the healthcare system has already proven to work wonders for patients. Artificial intelligence is a simulation of intelligence that uses data, rules, and information programmed in it to make predictions. The science of machine learning (ML) uses data to enhance performance in a variety of activities and tasks. A bigger family of machine learning techniques built on artificial neural networks and representation learning is deep learning (DL). To clarify, we require AI, ML, and DL to predict cancer risk, survival chances, cancer recurrence, cancer diagnosis, and cancer prognosis. All of these are required to improve patient's quality of life, increase their survival rates, decrease anxiety and fear to some extent, and make a proper personalized treatment plan for the suffering patient. The survival rates of people with diffuse large B-cell lymphoma (DLBCL) can be forecasted. Both solid and non-solid tumors can be diagnosed precisely with the help of AI and ML algorithms. The prognosis of the disease can also be forecasted with AI and its approaches like deep learning. This improvement in cancer care is a turning point in advanced healthcare and will deeply impact patient’s life for good.

## Introduction and background

Artificial intelligence (AI) is defined as algorithms or computer-coded programs that make decisions and predictions by analyzing the data, rules, and instructions programmed in them [1]. Another AI strategy is machine learning (ML), which is a program that teaches itself how to evaluate and understand data and detect patterns that even our brains are unable to recognize [1]. Artificial neural networks (ANNs) are software frameworks that draw inspiration from the biological neural networks seen in animal brains. Artificial neurons, which are a set of interconnected units or nodes that loosely resemble the neurons in a biological brain, are the foundation of an artificial neural network. Another algorithm, known as "deep learning (DL)," which is a type of machine learning, categorizes information similarly to how the human brain does. It employs techniques like artificial neural networks, which mimic how our neurons receive, process, and respond to information [1]. Cancer has always been a chronic, aggressive, and unpredictable ailment that has low survival rates depending upon the anatomical site and its staging. It is believed that precise and primary spotting of cancer is essential for the good projection of the disease, treatment, and survival of the patient [2]. Knowing who is more likely to get cancer in the future is necessary for early detection [3]. The art of forecasting the risk of having cancer is fortified by AI, ML, and DL. All these computer programs use data like a patient’s history, investigations done, scans taken, and other information to come to a conclusion of prediction or diagnosis or give a prognosis of the malignant illness [3]. The problems that stand in the way are varied. For instance, the involvement of AI in medicine has been restricted to some communities only and not always open to racial minorities or new kinds of patient populations [3]. Sometimes, it's also difficult and takes a lot of precision and years of experience to correctly diagnose various cancers and also differentiate cancers from benign growth or any other disorder. The prognosis prediction is also necessary to know as the treatment plan is broadly based upon it. Image-based risk models are being developed by researchers, but they must be validated through extensive scientific proof across several hospitals and computational advancement [3]. A factor model is a probability technique used to assign a person the probability that a future unfavorable outcome will occur over a particular time period [4]. Researchers say that tools like AI, ML, and DL are useful in the early prediction of cancer and its very essential for the good chances of cure and survival. Since aberrant DNA methylation could be a significant biomarker for tumor diagnosis, therapy, and prognosis, it is crucial to note that DNA methylation status is closely connected with a variety of disorders and is typically more stable than gene regulation [5]. The methylation of DNA regulates gene expression.

## Review

Objective

In this review article, we reviewed many pieces of research to know more about the parts of artificial intelligence (AI), machine learning (ML), and deep learning (DL) for the prediction, diagnosis, and prognosis of cancer. Our objective is also to discuss its shortcomings and other factors attached to the new technological advances and their adoption in the medical system.

Methodology

We searched the grand and prestigious medical database, PubMed, to find many articles about the topic “the role of AI, ML, and DL in the prediction, diagnosis, and prognosis of cancer”. We used the advanced search option using various keywords. Those keywords were "artificial learning," "machine learning," "deep learning," "cancer diagnosis and prognosis," "cancer prediction," etc. Among the various articles that were available, we selected articles from reputed journals like PubMed, Resuscitation, Springer Link, etc.

Discussion

**Figure 1:**
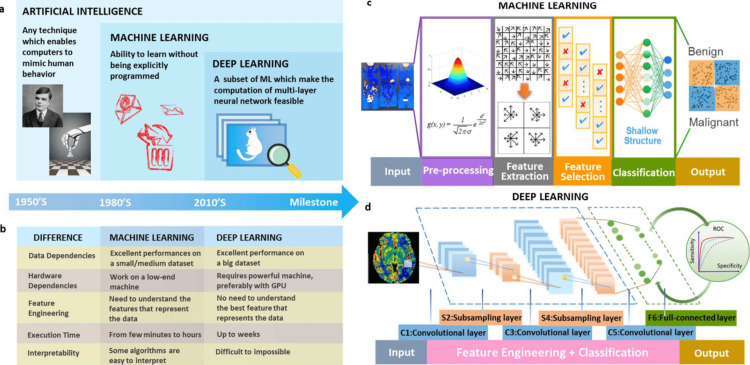
The ideas of AI, ML, and DL, as well as their connections, are shown in the figure. (a) AI is used to implement ML and DL, and DL is a technology that is utilized to execute ML. (b) Characteristics and distinctions between ML and DL in terms of feature engineering, processing speed, understandability, and reliance on hardware and data. (c) Machine learning relies on manufactured features that are taken from particular areas using specialized expertise. (d) Without using region annotations, DL employs localization for feature engineering. AI: artificial intelligence, ML: machine learning, DL: deep Learning. Open access journal under a CC-BY license contributed by Simon Fong, Qi Zhao, source [2].

In Figure 1, it is easier to understand the functioning and needs of AI, ML, and DL [2].

Fundamentals Around Artificial Intelligence

The idea of AI first originated in the mid-20th century as scientists sought to create machines that would exhibit the same cognitive abilities as humans. Artificial intelligence research is continuing to grow in many different areas, such as skilled methods, ML, progressive computing, logic, computed vision, regular language processing, and recommendation approaches. Essentially, ML uses algorithms to analyze data, learning the core patterns and providing information from which conclusions can be made and events in the real world can be predicted. Machine learning uses big chunks of details to "train" and use analysis to incrementally grasp how to accomplish specific functions. Deep learning is a dependent learning approach. Two types of "neural network training" methods are available: supervised and unsupervised. The exponential growth of this domain in the past has led to unique learning methods like residual networks. Thus, machine learning helps in appreciating artificial intelligence, while DL helps to implement it [2].

Machine Learning: Susceptibility Prediction 

An approach to objectively predict the occurrence of "spontaneous" breast cancer was developed in one of the research studies done by Listgarten et al. [6] in which they utilized single nucleotide polymorphisms (SNPs) profiles of steroid metabolizing enzymes (CYP450s). Ninety percent of all breast cancers are spontaneous or unrelated to families [7].

Machine Learning: Survival Prediction

To forecast the conclusion for sufferers with diffuse large B-cell lymphoma (DLBCL), a mixed ML technique was employed in one of the studies by Futschik et al. [8]. To be more precise, a single classifier for predicting the survival of DLBCL patients was created by combining clinical and genetic (microarray) data [7]. Generally speaking, a robust classifier is not necessarily ensured by a sample per feature ratio (SFR) of less than five [9].

*Machine Learning: Recurrence prediction* 

The research by De Laurentiis et al. [10] sought to predict, for patients with carcinoma (CA) breast, the likelihood of relapse throughout the entire period of five years. Scientific information, namely, the person’s age, the dimensions of the tumor mass, and the number of armpit lymph nodes transportation, were integrated with seven other anticipating factors. There were also details of protein biomarkers, such as levels of progesterone and estrogen receptors. The objective of the review is to create an automated, quantifiable prognostic technique that is comparatively more accurate than the conventional tumor-node-metastasis (TNM) staging algorithm. Some next-generation techniques like MammaPrint and Oncotype DX are very useful nowadays. MammaPrint is a prognostic and predictive diagnostic test that determines the likelihood that a tumor may spread to other areas of the body in individuals with early-stage breast cancer [11]. In people who will be undergoing hormone therapy for at least five years, the Oncotype DX test assesses the likelihood that breast cancer will metastasize within ten years of diagnosis [12].

*Using Technology in Colorectal Cancer Screening* 

By facilitating early detection and treatment and reducing colorectal cancer (CRC) incidence and death, screening aims to enhance patient prognosis [13]. Although endoscopy and fecal occult blood are common screening techniques, they have drawbacks. Numerous novel CRC screening prediction models, methodologies, and potential biomarkers have arisen as a result of the use of AI technology in the field of tumor screening. These developments are expected to increase the precision and lower the cost of CRC screening. A new sequencing technique called Cologuard testing is also very useful. The Cologuard test searches for DNA alterations that might be signs of colon cancer or precancerous polyps [14]. 

AI in the Diagnosis of Cancer

When assessing a patient's symptoms, physicians normally draw on their knowledge and professional experience. Diseases can be diagnosed using this clinical data and information, but there is no assurance that the detection and diagnosis will be accurate, and it is not possible to avoid incorrect diagnoses. This element illustrates how the brain's capacity to process enormous volumes of information is constrained. But the portrait of artificial intelligence is amazing at managing huge chunks of data. Because of the ability to study and teach big specimens, amalgamated preparation and exaction can result in more precise illness detection [2]. It is advised to integrate AI technologies with oncologic imaging, one of the primary techniques for cancer diagnosis. A group of computer models, namely deep learning, have lately been applied to achieve previously unheard-of improvements in the way computation devices exact data from photos. Many medical fields (most frequently radiology and pathology) have used deep learning algorithms to fulfill jobs, and in specific conditions, their work has been above the standard of human professionals. Additionally, it's conceivable that deep learning may aid in extracting information from clinical photographs that wouldn’t be evident at the moment by our analysis alone and could be utilized to provide information on the molecular phase of a patient, projection of disease, or therapy susceptivity [15].

Solid Tumor Diagnosis

Examining ultrasonographic images from ultra-sonography shows the adoption of a deep convolutional neural network (DCNN) model and that it can elevate the detection preciseness of thyroid malignancy [16]. The deep convolutional neural network model had comparable responsiveness and enhanced specificity in comparison to a team of knowledgeable radiologists, in detecting patients with thyroid cancer. The DCNN model's enhanced technical performance justifies additional research via clinical trials with randomization. According to Hu et al. [17], deep learning models can significantly affect clinical practice. With the help of the greatest number of images to date, a different study [18] constructed and verified, DCNN algorithms. The perfection in three small-scale validation sets, however, wasn’t adequate because it extends from a range of 0.857 to 0.889. Coudray et al. [19] programmed a DCNN to accurately and automatically identify pretty much the entire images using The Cancer Genome Atlas as lung adenocarcinoma (LUAD), lung squamous cell carcinoma (LUSC), or healthy part of the lung. The findings revealed that six of ten genes that are frequently mutated in LUAD may be predicted from pathological imaging, with area under the curve (AUC) figures varying from 0.733 to 0.856, as assessed by a hesitant group of people.

*Non-solid Tumor Diagnosis* 

For numerous guises of non-Hodgkin's lymphomas (NHLs), the detection of cluster and discriminant analysis indicates a merger of characteristics that are expanding rather than one is more predictive of distinguishing different lymphoma groups with disparate growth features in non-solid tumors [20]. The purpose of DL for the automated examination of H&E stained the detection challenge using histology pictures yielded an F-measure score of 5.06% and an increase in accuracy for the classification job of 1.09% [21]. Interestingly, a deep learning algorithm known as LYmph Node Assistant (LYNA), might identify progressive CA of the breast using biopsies of sentinel lymph nodes, increasing the fruitfulness of the doctor of pathology and lowering pseudo-rejection [22]. Haenssle et al. [23] contrasted 58 dermatologists to the convolutional neural network (CNN) in terms of diagnostic ability (30 of whom were experts).

AI in Clinical Prognosis Prediction of Cancer 

Clinical professionals of all kinds, from specialists to paramedics, have been asked to forecast cancer prognoses based upon their professional expertise over the past few decades. Clinicians recognize the significance of employing artificial intelligence technologies, such as ML and DL, as a conclusion to aid with the advent of the digital data era [2]. AI is also used to predict metastasis in the brain in one of the research [24]. It is challenging to forecast in which way cancer will develop given that conventional statistical analysis is unable to make reliable predictions. Clinicians are also worried about the possibility that a patient would get the disease, have a tumor reappear following therapy, or pass away. These factors have a significant impact on therapy options and therapeutic outcomes. In reality, the majority of current studies on clinical cancer focus on predicting how a patient will respond to treatment or determining the prognosis. More precise and effective treatments can be given to patients with more accurately predicted prognoses; in fact, these treatments frequently include individualizing or customizing care for each patient. In order to foresee cancer, AI can analyze and interpret multi-factor data from many patient evaluations and tell about the prognosis, patient survival, and the course of their disease more precisely [2]. To treat patients better, cancer prognosis includes knowing the higher probability of disease recurrence and patient survival [25,26]. Several methods were compared by Enshaei et al. [27], combining classifiers with regular old logistic regression analysis techniques to express that AI has a part in offering ovarian cancer patients forecasting and predicting information [28]. Khan et al. utilized various inference methods, membership functions, and decision tree rules for the analysis of survival rates of CA breast.

*Prognosis Prediction of Breast Cancer* 

Breast cancer prognosis incorporates estimating the likelihood of the illness returning and projecting the patient's survival, leading to better patient treatment. When integrating multi-dimensional data, researchers frequently employ multi-modal deep neural networks (DNNs) to the contrast between the receiver operating characteristic (ROC) curve and the area under the curve (AUC) values. The findings show that improving human breast cancer prognosis forecasts by merging various kinds of data and collection of DNN techniques is effective. The combination of multi-dimensional data, such as the portrayal of an expressed gene, copy number alteration (CNA) data, and medical database, to forecast the projection and human beings using multi-modal DNNs. A three-fold modal DNN and integrated anticipating scores from each separate non-dependent model make up the prediction model [29]. The outcomes showed that integrating various kinds of statistics with ensemble DNN techniques is an effective method to raise the accuracy of prognostic prediction for human breast cancer. In order to train artificial neural networks (ANNs) to recognize a framework for the categorization of recent occurrences, Jhajharia et al. [30] employed primary element analysis by early processing the data and bringing out characteristics in the most pertinent form. To better understand breast cancer survival in the context of absent data, Shukla et al. [31] built a robust data analysis approach. To analyze categorical variables and enhance prognosis utilizing classifiers, Abdikenov et al. [32] presented an N-N-based unit inserting strategy to obtain an uninterrupted bearing form of an unqualified changeable.

Prognosis Prediction of Gastric Cancer

In comparison to the Cox proportionate risk regression model, the ANN has been proven to be a greater effective analytical aid for forecasting the viability rate of patients. A survival recurrent network (SRN) was employed by Oh et al. [33] to forecast survival, and the outcomes were nearly correlated with actually surviving. Consequently, the SRN model performed better in predicting survival. In a different study [34], it was shown that the ANN model is a more effective method for identifying remarkable prognostic inconsistent figures for patients with gastric CA, which are encouraged for identifying their peril factors. Papp et al. [35] used Cox proportional hazard and ANN algorithms to examine 436 confirmed patients suffering from gastric cancer who had surgery at the Taleghani Hospital in Tehran (Iran) between 2002 and 2007 to estimate the survival time. In comparison to the traditional demographic approach (the Weibull regression model) [36,37], Mori et al. [17,38] demonstrated that the neural network model is an effective aid for identifying critical factors for people suffering from gastric cancer. By utilizing a nomogram obtained from the United States, a study [39] forecasted morbidity-specific cancer of gastric survival at an institute in Europe. The use of neural networks in the survival evaluation was compared to the Kaplan-Meier and Cox proportional risk images by Ciompi et al. [40].

## Conclusions

With the advancements in the technological world, AI-ML bloomed in this era. These new technologies were only limited to non-medical purposes but are beginning to get incorporated for the betterment of healthcare all over the world. One of the most devastating diseases, cancer, may have a cure shortly, but as prevention is better than cure, prediction of cancer, fast diagnosis, and prognosis prediction of the disease are crucial. The recent developments using AI-ML and deep learning are discussed in this review. The limitation of this paper is that we did not compare varied existing techniques to check cancer. The incorporation of AI, ML, and DL in the various types of cancer prediction, diagnosis, and prognosis can one day lead to a better solution for cancer. It can surely improve the quality of hospital settings as well, not just for cancer but for all ailments. The challenges related to this devastating ailment can surely be overcome, one day, by using these expanding algorithms.
